# Three dimensional structure directs T-cell epitope dominance associated with allergy

**DOI:** 10.1186/1476-7961-6-9

**Published:** 2008-09-15

**Authors:** Scott J Melton, Samuel J Landry

**Affiliations:** 1 Biomedical Sciences Graduate Program, Tulane University Health Sciences Center, New Orleans, LA, 70112, USA; 2 Department of Biochemistry, Tulane University Health Sciences Center, New Orleans, LA, 70112, USA

## Abstract

**Background:**

CD4+ T-cell epitope immunodominance is not adequately explained by peptide selectivity in class II major histocompatibility proteins, but it has been correlated with adjacent segments of conformational flexibility in several antigens.

**Methods:**

The published T-cell responses to two venom allergens and two aeroallergens were used to construct profiles of epitope dominance, which were correlated with the distribution of conformational flexibility, as measured by crystallographic B factors, solvent-accessible surface, COREX residue stability, and sequence entropy.

**Results:**

Epitopes associated with allergy tended to be excluded from and lie adjacent to flexible segments of the allergen.

**Conclusion:**

During the initiation of allergy, the N- and/or C-terminal ends of proteolytic processing intermediates were preferentially loaded into antigen presenting proteins for the priming of CD4+ T cells.

## 

CD4+ T-cell responses to dominant epitopes of protein allergens drive the development of allergic responses. CD4+ T-cells provide help to B cells that produce allergen-specific IgE, which is responsible for life-threatening anaphylactic reactions to allergens such as in insect venoms. Immunotherapy also depends on priming of CD4+ T-cells that either suppress the development of IgE-producing B-cells or help the development of IgG-producing B cells [1].

The epitope specificity of CD4+ T cells varies greatly among individuals; but when analyzed for a population, the dominance of certain epitopes becomes apparent, with some dominant epitopes recognized by a majority of subjects. The CD4+ T-cell epitope immunogenicity appears to have only a weak relationship to the composition of human leukocyte antigen (HLA) alleles in responding individuals [2-4]. Thus, CD4+ T-cell epitope dominance may be controlled at least in part by mechanisms of antigen processing.

Allergens taken up by professional antigen-presenting cells (APCs) are transported to an antigen processing compartment, where they or their peptide derivatives are loaded into class II major histocompatibility antigen-presenting proteins (MHCII) and proteolytically trimmed prior to display on the cell surface to T cells [5,6]. Acidification of the compartment modulates virtually all aspects of processing and presentation [7]. Acidification stimulates the activity of proteases responsible for antigen processing as well as maturation of the MHCII; it activates the peptide-exchange catalyst DM (also known as HLA-DM in humans) and the γ-interferon inducible lysosomal thiol reductase (GILT); and it destabilizes antigen structure. The degree of acidification depends on the type of antigen-presenting cell and degree of activation by danger signals, e.g., through Toll-like receptors.

Allergen/antigen structure has the capacity to modulate the accessibility of proteases and MHCII during antigen processing and presentation. Protease- and MHCII-binding sites form hydrogen bonds and other non-covalent contacts with allergen backbone and sidechain groups that, in the native allergen, stabilize three-dimensional structure. Low pH in the antigen-processing compartment is expected to destabilize the structure of allergens, but many proteins remain in a native or native-like conformation at low pH's [8,9]. The tendency for proteases to cleave initially in domain linkers and in flexible loops is well known, and the energetic penalty for unfolding 10–12 residues of polypeptide was found sufficient to explain the site selectivity of a serine protease acting on a native protein substrate [10]. The MHCII peptide-binding site envelopes an approximately 15-residue stretch of allergen [11], and thus MHCII binding to a structured protein is expected to involve an even larger energetic barrier than is involved in protease binding.

The dependence of CD4+ epitope immunogenicity on local structural context has been examined for a number of epitopes and antigens. Epitope presentation often may depend on an initial proteolytic-processing event. The engineering of a nearby dibasic protease-recognition sequence dramatically increased the presentation of an epitope in hen egg lysozyme [12]. The blocking of a cleavage site substantially reduced the overall immunogenicity of tetanus toxoid, presumably because the cleavage was necessary to globally unlock protein unfolding and further processing [13]. A more general demonstration of the relationship between structure and epitope dominance relies on the correlation between protease-sensitivity and conformational flexibility. The relative probability that a particular protein segment will be cleaved by a protease can be estimated by structural parameters that indicate conformational flexibility, such as crystallographic B factors, solvent-accessible surface area, or amide-group hydrogen/deuterium exchange (HX) [14-17]. Correlations of epitope dominance with one or more measures of flexibility has been reported for a number of antigens and allergens [18-22], but these studies have not identified the systematic exclusion of epitopes from the center of flexible segments in allergens, as is expected if proteolysis precedes MHCII binding.

This study analyzes the relationship of structure and CD4+ T-cell epitope dominance in the yellow jacket (wasp) venom allergen Ves v 5 and three additional unrelated allergens, and the results are interpreted in terms of a model for how allergen structure modulates allergen processing and epitope presentation.

## Methods

Epitope dominance was analyzed by converting peptide responses to a residue-by-residue *epitope score*, which corresponds to the average response to all peptides that contain the residue. In the study by Bohle et al., the specificity of T-cell lines or T-cell clones from 13 allergic individuals were mapped using a series of 12-mer peptides spanning the complete sequence of Ves v 5 with 9-residue overlaps [2]. This density of coverage results in exceptionally good resolution of epitope positions. Proliferative responses were considered positive when the stimulation index (SI) was greater than 4 for lines cultured from the peripheral blood mononuclear cells (PBMC) of allergic individuals. Epitope-mapping data for the other allergens were handled similarly, and relevant sources and features of the analysis are presented in Table 1.

**Table 1:** Allergen references and analytical  parameters.

**Allergen**	Ves v 5	Api m 1	Phl p 1	Cry j 1
aka	pathogenesis-related protein	phospholipase a2	expansin family	Polysaccharide lyase
Source	yellow jacket	honey bee	timothy grass	Japanese cedar
No. Residues	204	134	240	374

**Epitopes**				
No. Subjects	13	20	9	18
Peptide length	12	16*	12	15
Peptide overlap	9	10*	9	10
Step	3	8*	3	6
No. Peptides	65	19	76	69
Reference	[2]	[54]	[55]	[56]

**Corex stability**				
Structure file	1qnx	1poc	1n10	Cry j 1 model
Reference	[53]	[57]	to be published	[58]
Monte Carlo sampling	no	no	no	yes
Entropy scaling	0.994	0.885	0.85	1.039
Structure method	X-ray	X-ray	X-ray	X-ray
Homology model, template	no	no	no	yes, 1pxz

**B-factor**				
Structure file	1qnx	1poc	1n10	1pxz
Averaging window size	15	11	15	15
1st derivative averaging window size	9	11	9	9

**Sequence entropy**				
No. Homologs aligned	50	15	97	51
Range of identity	24–97%	11–77%	21–90%	10–75%
Strategy for identification	blast Genbank non-redundant	blast Swiss-Prot	blast Swiss-Prot	blast Swiss-Prot
Averaging window size	15	15	15	15
1st derivative averaging window size	9	9	9	9

*average value, peptide series not regular

Calculation of epitope scores, alignment of datasets, and analysis of correlation *vs*. offset were performed using Microsoft Excel. Significance tests were performed using GraphPad Prism. Residue-stability profiles were calculated using the COREX/BEST implementation at  with default values for all parameters [23]. Entropy factors were estimated with the COREX/BEST implementation, but they typically resulted in residue-stability profiles with less dispersion than could be obtained with a slightly lower entropy factor. Thus, the entropy factor was adjusted downward from the estimated value by 0.02. Residue solvent accessibilities were calculated using MOLMOL [24]. Crystal structures used for calculation of residue-stability and solvent accessibility are listed in Table 1. Since an high-resolution structure was not available for Cry j 1, an homology model was obtained on the basis of the structure of Jun a 1 (80% identical) using SwissModel [25]. For analysis of sequence entropy, homologous proteins were identified by the method noted in Table 1 and aligned using ClustalW [26]. Sequence entropy calculations were performed using BioEdit [27]. Significance of correlations was evaluated using t tests implemented in GraphPad Prism. In order to properly represent the sampling frequency of Ves v 5 epitope-mapping data, the significance tests were applied to paired datasets from which two-thirds of the points had been removed by retaining every third point.

For the identification of flexibility maxima in Ves v 5, profiles of B-factor, solvent-exposed surface area, and sequence entropy were smoothed with a 15-residue moving-window average. A "first-derivative" profile was generated by taking the difference between smoothed values for the current residue and the preceding residue. The first-derivative profile was smoothed with a 9-residue moving window average, and local maxima were assigned to residues where the smoothed first derivative became negative. For the identification of minima in COREX stability, a first-derivative profile was generated as described above using the raw COREX stability profile. The first-derivative profile was smoothed with a 7-residue moving window average, and local minima were assigned to residues where the smoothed first derivative became positive. Flexibility data for the other allergens were processed similarly, and the relevant sources and processing parameters are presented in Table 1.

## Results

### Consistent patterns of flexibility/stability

**Figure 1:**
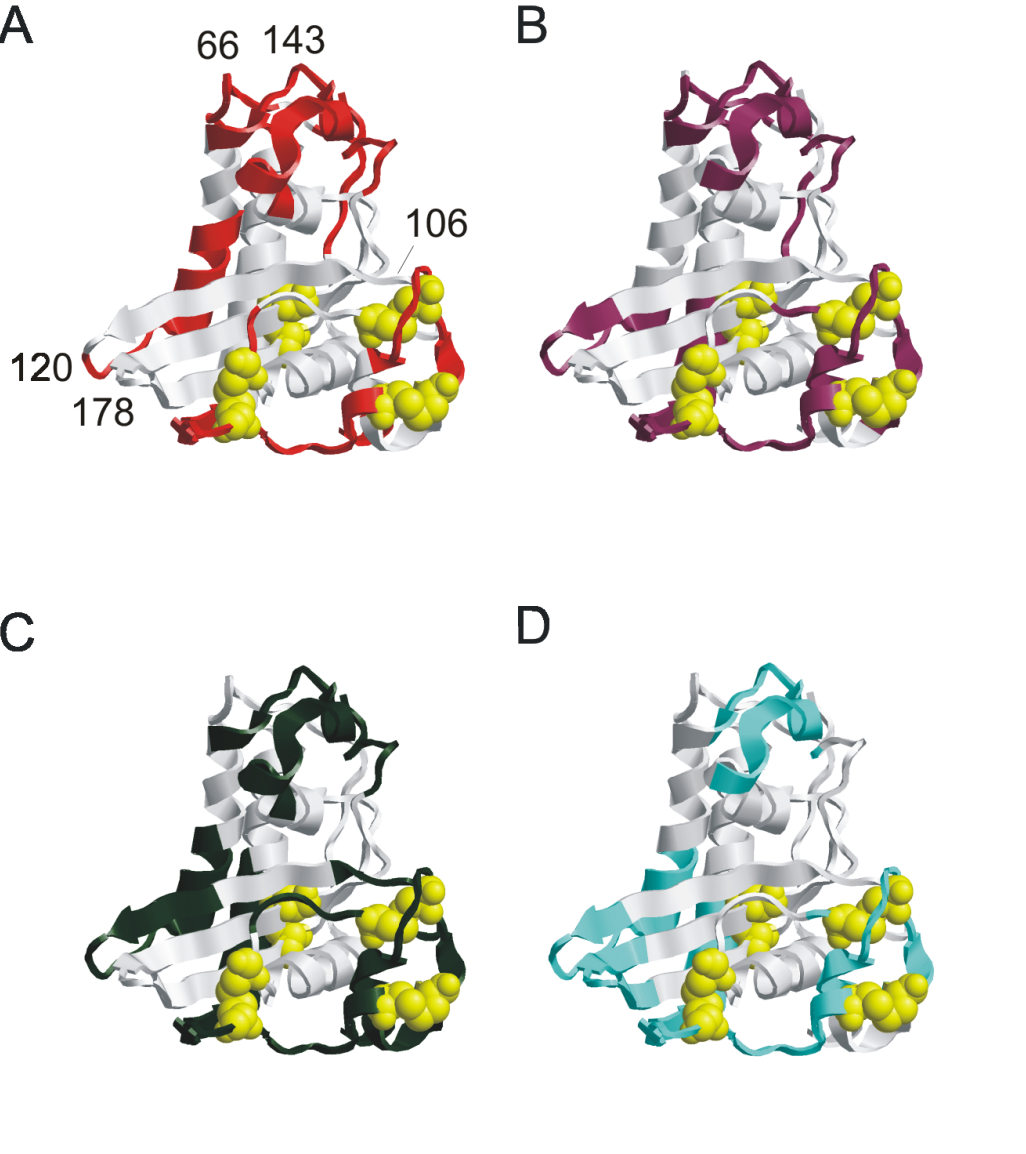
**Ribbon diagrams indicating flexible segments of the Ves v 5 crystal structure**[53]. Colored portions indicate regions of above-average flexibility as measured by crystallographic B-factors (A), solvent-accessible surface (B), COREX residue stability (C), and sequence entropy (D). Space-filled atoms correspond to disulfide-bonded cysteine residues. Numbers indicate the positions of residues near the centers of flexible sites.

Previous studies suggested that CD4+ epitopes tend to occur adjacent to proteolytic cleavage sites used during antigen processing [18-22]. Proteolytic cleavage sites were predicted on the basis of local conformational flexibility; and in some cases, proteolytic sensitivity was confirmed by limited proteolysis *in vitro *[28]. In order to identify probable sites of proteolytic cleavage in Ves v 5, the distribution of conformational flexibility in Ves v 5 was analyzed using three types of data based on the Ves v 5 crystal structure: the crystallographic B-factors for the backbone amide nitrogens, the fraction of amino-acid surface area that is solvent-accessible, and the COREX residue stability. Since flexible protein sequences tend to be poorly conserved [29], sequence entropy also was analyzed in a family of proteins homologous to Ves v 5. When mapped onto a ribbon diagram of the Ves v 5 crystal structure, it can be seen that the regions of high B-factor, high solvent-accessible surface area, low COREX stability, and high sequence entropy are located in similar regions of the protein. These regions are primarily in loops and turns between regular secondary-structure elements (Fig. 1).

### Allergenic epitopes on flanks of flexible segments

**Figure 2:**
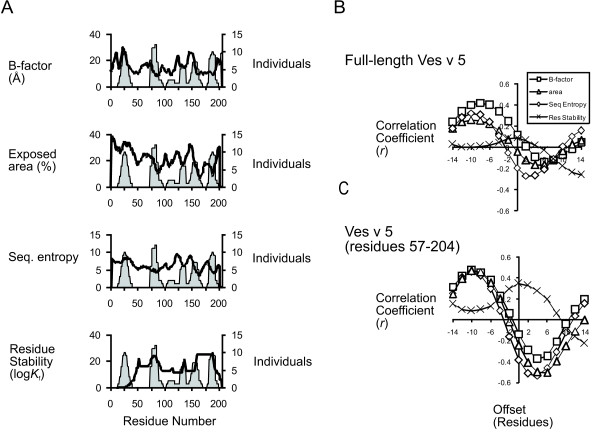
**Correlation of allergenic T-cell epitope dominance with adjacent segments of flexibility in Ves v 5**. In A, profiles of flexibility or stability (indicated by the line plots) are superimposed on the profile of epitope score (area plot), which is based on the T-cell responses reported by Bohle et al. [2]. In B, plots of correlation coefficient *vs*. offset for the full-length Ves v 5. In C, plots of correlation coefficient *vs*. offset for the protein N-terminally truncated at residue 57.

When aligned with the profile of epitope score for allergic individuals, the various profiles of flexibility reveal a number of peaks or valleys (five to seven) that is similar to the number of peaks of epitope score (six). As has been noted previously for other antigens, some Ves v 5 epitopes occur on the flanks of peaks of flexibility. For example, the peaks of epitope score near residues 125 and 150 occur C-terminal from adjacent flexibility maxima and N-terminal from adjacent flexibility minima, and the pattern of flexibility in these regions of Ves v 5 is consistently reported by all four measures of flexibility/stability (Fig. 2A).

The peak of epitope score near residue 25 is located in a large N-terminal segment of irregular structure that is characterized by partially overlapping peaks of flexibility and a broad region of very low COREX residue stability. Rather than being located on the flank of a well-defined maximum of flexibility, this peak of epitope score is located in the middle of a large flexible N-terminal region of the protein. This region most likely is ordered in the crystal structure because two disulfide bonds stabilize it, but it could easily be disordered in mildly denaturing conditions or after cleavage of the disulfide bonds (which may occur in an antigen-processing compartment).

In order to quantify the strength of the relationship between epitope dominance and conformational flexibility and to investigate the mechanism, the profiles of flexibility/stability and epitope score were tested for correlation at various offsets of one dataset to the other. The procedure effectively tests for correlations between epitopes and flexibility in nearby N-terminal or C-terminal segments. Plots of correlation vs. offset illustrate a transition from positive on the left to negative on the right, indicating that epitope dominance correlates with N-terminal flexibility and C-terminal stability in the adjacent sequences (Fig. 2B). The maximum correlations (or anti-correlations) and offsets are as follows: B-factor, 0.42 at offset = -8; solvent-exposed area, 0.32 at offset = -6; and sequence entropy, 0.26 at offset = -6. The correlation of epitope score and residue stability did not achieve significance (p < 0.05) over the range of offset tested. Thus, for three out of four flexibility criteria, optimum correlations were obtained at similar values of offset. These initial results suggested that epitopes occur 6 to 8 residues C-terminal from flexible sites.

In the course of the analysis, it became clear that the correlation of epitope score with flexibility breaks down in the N-terminal irregularly structured region of Ves v 5; and thus the correlations were reevaluated for a portion of the protein lacking this segment (Fig. 2C). For Ves v 5 truncated at residue 57, significant correlations and significant anti-correlations with flexibility were observed for optimum values of negative and positive offset, respectively. At negative offset, correlations and offsets are as follows: B-factor, 0.47 at offset = -10; solvent-exposed area, 0.47 at offset = -10; and sequence entropy, 0.46 at offset = -10. At positive offset, anti-correlations were as follows: B-factor, -0.37 at offset = 4; solvent-exposed area, -0.54 at offset = 4; sequence entropy, -0.51 at offset = 6. The correlation with COREX residue stability achieved a maximum of 0.35 at offset = 0. Thus, in the analysis of Ves v 5 residues 57–204, correlations were obtained with all four flexibility criteria, and the correlations were stronger. The values of offset suggest that epitopes occur 10 residues C-terminal from flexible sites and 4–6 residues N-terminal from stable/inflexible sites. The correlation with residue stability suggests that epitopes occur right on top of the stable/inflexible sites.

### Exclusion of allergenic epitopes from flexible segments in a selection of well-characterized allergens

Although significant correlations were occasionally observed in other allergens, the correlation coefficients were small (|r| < 0.25). In some cases, correlations exhibited two maxima, one on each flank of the flexible site (data not shown). As we have noted previously in surveying CD4+ epitope maps [30], epitopes tend to occur on either the N- or C-terminal flank of flexible sites. In Ves v 5 the C-terminal flanks are preferentially loaded, but in other antigens/allergens the preference is opposite, or there is a mixture of the two. When both flanks are utilized in the same antigen/allergen, any correlation that uses a particular offset has a low correlation coefficient.

**Figure 3:**
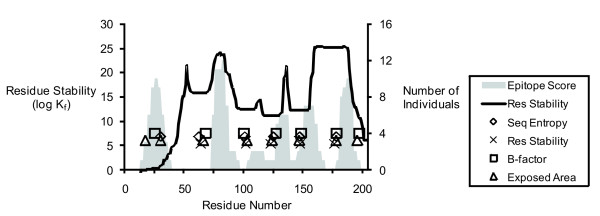
Localization of T-cell epitopes between peaks of conformational flexibility in Ves v 5.

The tendency for epitopes to be localized adjacent to flexible sites suggests that they are less likely to occur *within *the flexible site. In order to visualize the relationship of flexible segments and dominance peaks, the flexibility maxima (or stability minima) were plotted as single points on a graph of epitope score in allergic individuals. In this illustration, epitopes appear to be excluded from segments of maximum flexibility/instability, with the exception of the dominance peak at residue 25 (Fig. 3).

In order to demonstrate the generality of this pattern, the same analysis was applied to the bee venom allergen, Api m 1, and pollen allergens, Phl p 1 (Timothy grass) and Cry j 1 (Japanese cedar).

**Figure 4:**
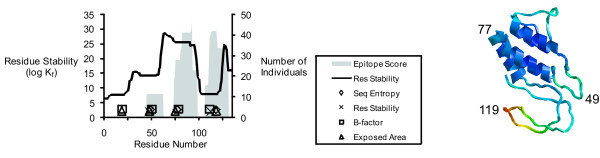
**Localization of T-cell epitopes between peaks of flexibility in Api m 1 and the ribbon diagram of the Api m 1 crystal structure.** The ribbon diagram is colored by B-factor (red, high; blue, low), and numbers indicate the positions of residues near the centers of flexible sites.

For Api m 1, the maxima from the flexibility profiles yielded very tight clusters (Fig. 4). Two of the clusters coincided with minima in the residue-stability profile (near residues 49 and 119). A third cluster defined by B-factor, solvent-accessible area, and sequence entropy was located in the center of a broad peak of residue stability (near residue 77). Thus, it is clear that reliance on residue stability for identifying flexible sites may miss some of them, and the combination of criteria provides a robust strategy for identifying flexible sites. In Api m 1, the three prominent flexible sites were each associated with a gap in the T-cell response on the N-terminal side and a peak of T-cell response on the C-terminal side, which is similar to the pattern observed for Ves v 5.

**Figure 5:**
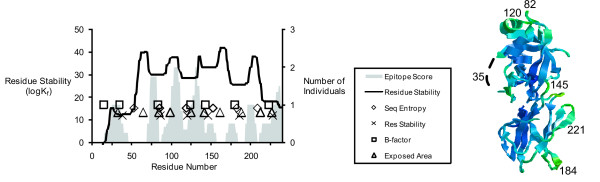
Localization of T-cell epitopes between peaks of flexibility in Phl p 1 and the ribbon diagram of the Phl p 1 crystal structure (as described in Fig. 4, legend).

In Phl p 1, the three most allergenic segments were N-terminally adjacent to tightly clustered maxima near residues 82, 120, and 145 (Fig. 5). The next most allergenic segments are C-terminally adjacent to flexible sites near residues 184 and 221. The remaining allergenic segments were associated with flexible sites that partially overlap the epitopes. The N-terminal allergenic segment is centered on a segment (near residue 35) that is so flexible that it was disordered in the crystal structure. Two other weakly allergenic segments were associated with flexible segments (near residues 145 and 184) that lie between major domains or subdomains.

**Figure 6:**
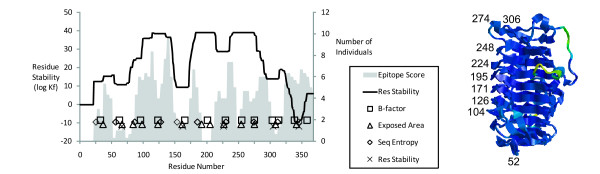
Localization of T-cell epitopes between peaks of flexibility in Cry j 1 and the ribbon diagram of the crystal structure for the Cry j 1 homolog, Jun a 1 (as described in Fig. 4, legend).

In Cry j 1, the flexibility minima are well-clustered in regularly-spaced groups (Fig. 6). Peaks of epitope dominance were located neatly between the clusters.

## Discussion

The pattern of CD4+ epitope dominance that was observed for Ves v 5 in allergic individuals supports the hypothesized relationship to allergen structure. The number of allergenic epitopes is similar to the number of peaks of structural flexibility or stability, and the epitopes tend to be situated between the flexible and inflexible regions. The most frequent spacing of epitopes between flexible and inflexible regions (10 residues C-terminal from a flexibility maximum and 4–6 residues N-terminal from a stability maximum) is remarkably similar to the spacing of 12 residues C-terminal from the flexibility maximum that was described for T-helper epitopes in the outer domain of HIV gp120 [31].

The spacing observed in Ves v 5 is different from that observed in influenza hemagglutinin, wherein epitopes most frequently occurred 10 residues C-terminal from the *stability *maximum, rather than the flexibility maximum [30]. However, the pattern in hemagglutinin was a mirror-image of the pattern in Ves v 5 and gp120. In hemagglutinin, epitopes were on the N-terminal side of flexible segments, rather than the C-terminal side. For all three antigens/allergens, epitopes tended to be excluded from the most flexible sites in the proteins.

In the following discussion, patterns of epitope dominance will be discussed in the context of a mechanism by which allergen structure directs CD4+ T-cell epitope dominance. The mechanism is hypothesized to have the following elements:

1. Epitope dominance is due to preferential presentation of certain peptide-MHCII complexes. Although at least one report argues that the abundance of epitope presentation has little influence on immunodominance [32], other studies suggest that antigen processing and presentation have a potent influence on immunodominance [19,33,34]. We take the position that, especially in regard to promiscuously dominant epitopes (which are presented by more than one allele of MHCII), the cause of the dominance is the preferential presentation of the epitope.

2. Proteases and MHCII co-mingle in the antigen-processing compartment and therefore can compete for the sequences that satisfy requirements for binding to both proteases and MHCII. The following observations support this postulate. Several proteases are implicated in the processing of both antigens and the MHCII-bound invariant chain [35-37]. The proteolytic separation of two MHCII-bound epitopes was found to be a rate-limiting step in presentation of the epitopes [38]. The level of activity of asparagine endopeptidase (AEP) can control the presentation of an epitope that contains a cleavage site for AEP [39].

3. The antigen/allergen remains in a native-like conformation through the initial proteolytic nicking of the protein and/or loading of a fragment into an MHCII. Studies of protein folding and stability have provided examples in which proteins retain elements of native-like structure at low pH [9], following proteolytic nicking [40,41], and when parts of the protein are demonstrably unfolded [42,43].

4. Proteases and MHCII preferentially bind to antigen/allergen sequences that have low conformational stability and adequate affinity for the binding site. To some extent, these two properties could be mutually exclusive. For example, hydrophobic sidechains can stabilize binding of an antigen segment to both proteases [44] and MHCII [45,46], but hydrophobic sidechains also tend to be buried in structurally stable elements of protein structure [47], where they are unavailable for binding. Thus, there exists a three-way competition for interactions with antigen/allergen sequences that involves intramolecular folding, binding to the protease, and binding to the MHCII.

5. Proteases bind shorter flexible segments of antigen/allergen than MHCII [11,48]. It follows that the on-rates of proteases are faster than the on-rates of MHCII because the smaller binding sites require less reordering of the polypeptide and because short flexible segments occur with higher frequency than the long flexible segments in the natively folded antigen/allergen.

6. MHCII bind stably to epitopes that have adequate binding affinity. Thus, the MHCII protect the bound segments from proteolysis [49]. However, the kinetics of MHCII binding and dissociation are modulated by DM, which responds to APC activation [7,50].

7. The extensive and stable interactions of antigen/allergen segments with the MHCII provide a driving force for unfolding the antigen. Although we are unaware of any direct evidence supporting this postulate, peptide binding to MHCII has been described as cooperative process akin to protein folding [51]. In the absence of coupling to any energy source, the assembly of the peptide-MHCII complex and disassembly of the antigen/allergen structure may be considered two sides of a thermodynamic equilibrium.

**Figure 7:**
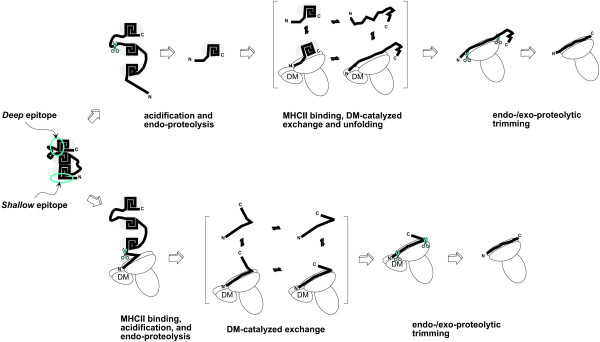
**Models for Ves v 5 processing and the presentation of allergenic and epitopes.** Stable protein segments are shaded. For clarity, only three stable segments are illustrated. Presentation of the deep epitope (upper pathway) requires an initial cleavage that yields a proteolytic fragment whose N-terminal end binds to the MHCII. In the course of DM-catalyzed dissociation and rebinding, the fragment unfolds and optimizes interaction with the MHCII binding site. Presentation of the shallow epitope (lower pathway) is similar; except that an initial cleavage is not required before MHCII binding, and no significant unfolding occurs during DM-catalyzed dissociation and rebinding. Proteolytic trimming of the MHCII-bound fragments yield MHCII-peptide complexes that traffic to the surface of the APC.

These elements have been incorporated into a model for processing and presentation of allergenic epitopes (Fig. 7). The model illustrates the presentation of two epitopes but it is not intended to suggest that both epitopes are presented from every molecule, nor is it to suggest that other epitopes would not also be presented. These two epitopes are examples of two types, "deep" and "shallow", which are distinguished by the order of proteolysis and peptide loading. In the upper pathway, proteolysis precedes loading of the "deep" epitope. In the lower pathway, loading of the "shallow" epitope precedes proteolysis. Although it is possible that an antigen/allergen would be cleaved at multiple sites and adjacent epitopes be presented from each fragment, the fact that most allergic individuals mount a response to only one or two epitopes suggests that the more likely scenario is the presentation of one or two epitopes after a single proteolytic event. Subsequent proteolytic cleavages may produce fragments that are too small and unstable to resist complete proteolytic degradation.

Six of seven peaks of epitope dominance in Ves v 5 lie adjacent to peaks of flexibility. In the model, this pattern of epitope dominance is shown to arise from an initial endoproteolytic nick in a flexible segment, followed by loading of an adjacent epitope in the MHCII (Fig. 7, upper pathway). The regularity of this relationship is highlighted in the plot illustrating the flexibility maxima as single data points (Fig. 3). These six epitopes are effectively excluded from the most flexible regions, and they overlap the least flexible regions. Since these epitopes include residues that are buried in the protein interior, they are called "deep" epitopes. These epitopes are partially buried or otherwise sequestered from MHCII binding by three-dimensional structure until proteolytic nicking at a nearby site renders the epitope more accessible.

The dominance peak at residue 25 of Ves v 5 is exceptional in that it lies squarely over a flexibility maximum as defined by several criteria. This "shallow" epitope requires no proteolysis and little unfolding for loading into the MHCII. The dominance of this epitope probably results from a combination of good flexibility/accessibility and protection from proteolysis by continued association with the MHCII through multiple cycles of DM-catalyzed dissociation and rebinding (Fig. 7, lower pathway).

The positions of epitopes in venom allergen Api m 1 and pollen allergens Phl p 1 and Cry j 1 were not so regularly spaced from a maximum or minimum of flexibility that significant correlations could be identified at a single offset (data not shown). Nevertheless, most of the epitopes were excluded from the center of flexible segments. This pattern is consistent with cleavage of these allergens in the flexible regions, followed by loading into the MHCII of the newly generated fragments. Apparent exceptions to this general trend are discussed in the following.

All three dominance peaks in Api m 1 overlap the adjacent peaks of flexibility, which would seem to be incompatible with the "proteolysis-first" mechanism. However, as for most antigens/allergens, the naturally-processed MHCII ligands for Api m 1 have not been characterized, and thus we do not know the exact position of the N-termini. In the available study of Api m 1, epitopes were mapped using an irregular series of peptides that spanned the sequence in an average of 8-residue steps. Thus, it is possible that that the epitopes could be refined to smaller sequences whose N-terminal ends coincide with the most flexible, protease-sensitive sites, which would be completely consistent with the "proteolysis-first" mechanism.

For Phl p 1, the epitope(s) near residue 35 seems to be a strong candidate for the "binding-then-proteolysis" pathway because the epitope is centered on a 12-residue segment that was disordered in the protein crystal. Presumably, this segment has good affinity for the MHCII; and therefore it resists DM-catalyzed dissociation from the MHCII and is protected from proteolysis. The weakly immunogenic epitopes near residues 145 and 184 coincide with regions of flexibility, as defined by all four criteria. The modest immunogenicity of these flexible regions could be related to their location near the N-terminus of the second major domain of Phl p 1. An initial cleavage on the N-terminal side of the epitope at residue 145 may yield an independent molecule. N-terminal disordered segments may be particularly good ligands for MHCII. This hypothesis is consistent with the exceptional immunogenicity of the N-terminal epitope in Ves v 5 and Phl p 1, and it is also consistent with the regular pattern of epitopes on the C-terminal flank of flexible sites in Ves v 5 and HIV gp120, which suggested that the MHCII binds near the N-terminus of proteolytic fragments.

The regular pattern of epitope dominance in Cry j 1 is strikingly similar to the regular pattern of flexible sites in that protein. In the ribbon diagram, the basis for the patterns is apparent in the regular turns of the pectin-lyase-like beta helix. Turns of the beta helix recur at an average interval of 29 residues, and the flexible sites are distributed in a stripe down one face of the beta helix. Presumably, any one of these flexible sites can serve as the initial site for proteolytic cleavage, followed by loading of an adjacent epitope.

The bias toward dominance of epitopes on the C-terminal side of flexible sites in Ves v 5 suggests that a feature of either the loading mechanism or the structure of the protein favors loading of an N-terminal sequence after an initial proteolytic cleavage. However, the number of epitopes illustrating this bias constitutes too small of a sample to establish such a relationship. Although the epitopes tend to be closer to flexible sites on the C-terminal side, their positions are nearly centered on the stable/inflexible segments, as indicated in Ves v 5 by the anti-correlations with flexibility at offsets of 4–6 residues and the correlation with COREX residue stability at zero offset. In a compiled analysis of epitope spacing in nine antigens/allergens, epitopes were found to occur with equal frequency on the N-terminal and C-terminal sides of flexible sites [52]. On the basis of the data available, the existence of a bias toward loading the N-terminal or C-terminal fragment in any given antigen/allergen must be regarded as anecdotal.

Although many details of the allergen processing and presentation mechanism remain to be elucidated, the exclusion of epitopes from flexible sites in allergens suggests that the three-dimensional structure of the allergen exerts a strong influence on the pattern of CD4+ epitope dominance by targeting the initial sites of proteolytic processing.

## Competing interests

The authors declare that they have no competing interests.

## Authors' contributions

SJM participated in the analysis of Ves v 5 structure, the correlation of structural features with epitope dominance, and drafted, read, and approved the manuscript. SJL conceived of the study, and participated in its design and coordination and drafted, read, and approved the manuscript.
